# Dataset for Sun dynamics from topological features

**DOI:** 10.1016/j.dib.2023.109728

**Published:** 2023-10-26

**Authors:** M. Tarazona-Alvarado, D. Sierra-Porta

**Affiliations:** a Universidad Industrial de Santander, Escuela de Física. Car 27 #9, Bucaramanga, 680001, Santander, Colombia; b Universidad Tecnológica de Bolívar, Facultad de Ciencias Básicas, Parque Industrial y Tecnológico Carlos Vélez Pombo Km 1 Vía Turbaco, Cartagena de Indias, 130010, Bolívar, Colombia

**Keywords:** Sun´s dynamics, Spectral features, Image processing, Space weather

## Abstract

The present study presents an extensive dataset meticulously curated from solar images sourced from the Solar and Heliospheric Observatory (SOHO), encompassing a range of spectral bands. This collaborative effort spans multiple disciplines and culminates in a robust and automated methodology that traverses the entire spectrum from solar imaging to the computation of spectral parameters and relevant characteristics.

The significance of this undertaking lies in the profound insights yielded by the dataset. Encompassing diverse spectral bands and employing topological features, the dataset captures the multifaceted dynamics of solar activity, fostering interdisciplinary correlations and analyses with other solar phenomena. Consequently, the data's intrinsic value is greatly enhanced, affording researchers in solar physics, space climatology, and related fields the means to unravel intricate processes.

To achieve this, an open-source Python library script has been developed, consolidating three pivotal stages: image acquisition, image processing, and parameter calculation. Originally conceived as discrete modules, these steps have been unified into a single script, streamlining the entire process. Applying this script to various solar image types has generated multiple datasets, subsequently synthesised into a comprehensive compilation through a data mining procedures.

During the image processing phase, conventional libraries like OpenCV and Python's image analysis tools were harnessed to refine images for analysis. In contrast, image acquisition utilised established URL libraries in Python, facilitating direct access to original SOHO repository images and eliminating the need for local storage.

The computation of spectral parameters involved a fusion of standard Python libraries and tailored algorithms for specific attributes. This approach ensures precise computation of a diverse array of attributes crucial for comprehensive analysis of solar images.

Specifications Table

**Table:** 

Subject:	Space weather
Specific subject area:	Data Science, Heliosphere, Sun dynamics, Topology, Time series
Data format:	Raw and Filtered
Type of data:	Text Files (csv-formated)
Data collection:	Solar disk images obtained from SOHO space satellite. To obtain our data, we have taken the public SOHO images and written a Python code to calculate (from each image), various indices and spectral features from the time series. The Python codes are also publicly available at the web address: https://soho.nascom.nasa.gov/data/REPROCESSING/Completed/.
Data source location:	Public open data from SOHO space mission. URL: https://soho.nascom.nasa.gov/data/REPROCESSING/Completed/. Public open data from SILSO (Royal Observatory of Belgium) Brusells Observatory. URL: https://www.sidc.be/SILSO/datafiles
Data accessibility:	Repository name: Mendelay Data Data identification number: 10.17632/5gh3xbvc92.1 Direct URL to data: https://data.mendeley.com/datasets/5gh3xbvc92/1

## 1 Value of the Data

• In the realm of heliospheric and solar investigation, the acquisition of robust and comprehensive data holds paramount significance in unraveling the underlying mechanisms governing solar activity. The current endeavor, centered on curating time-series datasets through solar images captured by the Solar and Heliospheric Observatory (SOHO) [1], presents an opportunity to expand our comprehension of solar dynamics and its implications within the interplanetary space milieu. As the scientific community delves into inquiries surrounding the nuances of solar activity and its ramifications at terrestrial and cosmic levels, this dataset emerges as a pivotal resource for advancing our understanding within this domain of study.
• The unique essence of this project lies in its multidimensional approach, amalgamating spectral data and topological attributes extracted from solar images across a spectrum of light ranges, spanning from visible to ultraviolet and infrared. These images encapsulate a substantial reservoir of information pertaining to solar activity across varying temporal scales, encompassing phenomena from sunspots to solar flares. By computing diverse indices and features that encapsulate the images' intensity, texture, and intricacy, this dataset crystallizes as an inherent trove of insights into solar behavior. These images encapsulate a substantial reservoir of information pertaining to solar activity across varying temporal scales, encompassing phenomena from sunspots to solar flares. By computing diverse indices and features that encapsulate the image's intensity, texture, and intricacy, this dataset crystallizes as an inherent trove of insights into solar behavior.
• The substantial potential of this dataset extends beyond the confines of its immediate application in modeling specific solar traits. Within these supplementary attributes, the scientific community will discover an invaluable resource nurturing the formulation and refinement of predictive and explanatory models for intricate solar phenomena, including fluctuations in magnetic activity and interplanetary oscillations. The correlation and evaluation of these attributes in relation to other solar parameters establish a critical pathway towards a more enriched comprehension of the interlinkages among disparate facets of solar activity, thereby shedding crucial light on predicting potentially disruptive space weather events within our solar system's framework.
• The accessibility of such an expansive and meticulously computed dataset will not only augment individual research endeavors but will also establish a platform for the flourishing of collaboration and the exchange of ideas within the scientific realm. Researchers hailing from diverse disciplines, spanning from solar physics to astrophysics and space climatology, will find within this dataset an irreplaceable asset for substantiating and refining their theoretical propositions and models. The convergence of multidisciplinary data might unveil patterns and trends that have hitherto remained concealed, unveiling novel avenues for comprehending solar processes and their repercussions across the solar system in its entirety.
• The dataset generated from these solar images and their spectral characteristics has crucial applications in the training of prediction and forecasting models in multiple contexts. On the one hand, it allows the development of solar flare forecasting models by analyzing the images and associated features, which is essential to ensure the safety of space missions and the protection of infrastructures on Earth. In addition, these features can be used to model fluctuations in the solar magnetic field, which is relevant for understanding and anticipating changes in the Earth's magnetosphere and their impact on navigation and communications. Finally, the dataset is also valuable for investigating and predicting patterns in long-term solar cycles, contributing to the understanding of solar activity over time and its influence on space weather. These applications are critical for astronaut safety, space mission planning, and protection of ground-based infrastructure from potentially disruptive solar events.

## 2 Data Description

We have generated an impressive and diverse dataset from time series, encapsulating information from six distinct filters of the SOHO cameras. These data span both the native image resolution and capture frequency, providing a detailed and temporally rich view of solar activity. Notably, the HMIIGR and HMIMAG (Helioseismic and Magnetic Imager Intensitygram) in the electromagnetic spectrum range of the visible region and part of the near-infrared spectrum, respectively, filters offer captures every hour and a half [2,3], and EIT171, EIT195, EIT284 and EIT304 (Extreme Ultraviolet Imaging Telescope 304) in the extreme ultraviolet region, for 171, 195, 284 and 304 Å, respectively provide two images per day [4,5].

This wide range of capture frequencies allows for a deep exploration of patterns and changes in solar activity across different temporal scales. Additionally, the computation of 14 key parameters in the images, such as: entropy, mean intensity, standard deviation, skewness, kurtosis, relative smoothness, uniformity, fractal dimension, taruma contrast, taruma directionality, taruma coarseness, taruma linelikeness, taruma regularity and taruma roughness, adds a significant dimension to our data [6]. These parameters offer a detailed characterization of the properties of solar images, from texture to complexity and regularity.

**Fig. 1:**
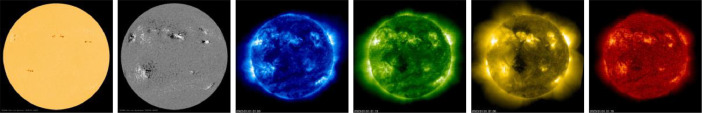
Images used for the construction of the results. All images are for day 20230101 and are presented in order from left to right HMIIGR and HMIMAG (Helioseismic and Magnetic Imager Intensitygram) in the electromagnetic spectrum range of the visible region and part of the near-infrared spectrum, respectively, and EIT171, EIT195, EIT284 and EIT304 (Extreme Ultraviolet Imaging Telescope 304) in the extreme ultraviolet region, for 171, 195, 284 and 304 Å, respectively.

The chosen time span, from 2011 to the present year, is highly relevant as it encompasses a significant range of changes in solar activity and associated phenomena. This enables the observation of long-term trends, solar cycles, and anomalous events that might have occurred during this period (See Fig. 1).

Data hold immense potential for delving into the understanding of solar activity in its multiple dimensions. From the differences in resolution and capture frequency to the richness of the calculated parameters, your work provides a solid foundation for interdisciplinary research in fields like solar physics, space climatology, and image analysis. These data could be key to uncovering correlations and patterns not previously evident, potentially leading to new insights into solar dynamics and its implications in interplanetary space. In summary, your dataset represents a valuable contribution to knowledge in the field of solar research and its impact on the surrounding space.

## 3 Experimental Design, Materials and Methods

For the purpose of creating this database, we initially developed an open-source Python code library script. This initial library script served to retrieve the images from server (https://soho.nascom.nasa.gov/data/REPROCESSING/Completed/). Subsequently, we crafted another Python code library to handle the image processing phase. Lastly, a final library was created to compute the parameters and spectral characteristics. All these individual libraries were amalgamated into a comprehensive script that performs all the necessary tasks automatically.

To produce the various datasets, we execute the main script for each image type. Following this, a data mining process is employed to consolidate the obtained data into the ultimate dataset. Notably, during image processing, we rely on commonly used and standard OpenCV libraries along with Python's image analysis capabilities. In contrast, for image retrieval tasks, established URL libraries are utilized. It's important to highlight that throughout this process, images are not downloaded to the hard disk. Instead, the entire workflow revolves around accessing the images directly from the original SOHO repository.

Lastly, when it comes to calculating spectral parameters, a combination of standard Python libraries is employed for certain aspects. However, for other specific features, custom proprietary code has been developed.

**Fig. 2:**
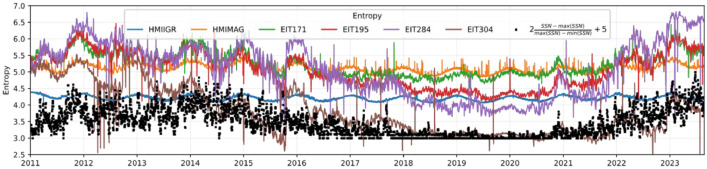
Entropy variations observed across different filtered image series obtained from diverse filters. The *x*-axis represents the chronological progression of time, while the *y*-axis displays the entropy values. Each line on the graph corresponds to a distinct filter, revealing the dynamic changes in image complexity over time.

The dataset comprises 14 meticulously calculated parameters, each offering a distinctive mathematical insight into the solar images. These parameters encompass a spectrum of characteristics that span the complexity, texture, and intensity of the images. Entropy quantifies the image's randomness and diversity, such as $E\;=\;-\;\sum_{i=1}^{N}p_{i}\;.\;log_{2}\left(p_{i}\right)$ where $p_{i}$ represents the probability of occurrence of a particular pixel intensity value within the image. It's a measure of how frequently a certain intensity value appears in the image. Mathematically, $p_{i}$ is calculated by dividing the number of occurrences of intensity value by the total number of pixels in the image N. This probability is then used in the entropy formula to compute the entropy of the image based on the distribution of pixel intensity values. From Fig. 2 we can see that entropy parameter is very correlated with sunspot number in same interval more visually impacting in the extreme ultraviolet filters.

The mean intensity (μ) is calculated by summing up the product of pixel intensities and their corresponding probabilities, which is essentially the average intensity value in the image. Standard deviation (σ) is calculated using the probabilities and pixel intensities. It quantifies the dispersion of pixel intensities from the mean intensity. Skewness (g) measures the asymmetry of the intensity distribution. The code calculates it using the probabilities and intensities, focusing on how the distribution deviates from symmetry. Kurtosis (K) quantifies the peakedness of the intensity distribution. Similar to skewness, it's calculated using probabilities and intensities, assessing how the distribution deviates from a normal distribution.

Uniformity represents how even the distribution of pixel intensities is. It's calculated by summing the squared probabilities. Relative smoothness is calculated using the standard deviation, with a formula that transforms it into a value between 0 and 1, indicating the level of smoothness or roughness in the image. The Taruma Contrast metric is designed to assess the contrast characteristics of an image based on its standard deviation and kurtosis. These statistical properties provide insights into the distribution of pixel intensities and their relative frequency. The Taruma Contrast is computed using $C_{taruma}\;=\;\sigma^{2}/\;K^{0.25}.\;$The Taruma Contrast metric reflects the balance between the image's standard deviation and kurtosis, providing a measure of how the distribution's shape affects the perceived contrast. A higher value indicates a stronger contrast between pixel intensities, while a lower value suggests a more balanced or distributed intensity range. This metric is valuable for characterizing the image's contrast properties and can be applied in various image analysis tasks, including texture analysis, quality assessment, and image enhancement.

The Taruma Directionality metric quantifies the predominant directionality of features within an image. It involves analyzing the gradients and direction of edges to determine the overall directionality of patterns present in the image. The Taruma Directionality is computed using $D_{tatima}=\;1\;-\;\left(r.n.F_{dir}\right)$, where r is a normalization factor, n is the number of bins in the directionality histogram, and $F_{dir}$ represents a measure of the distribution of directions in the image. The calculation of $F_{dir}$ involves constructing a directionality histogram based on the gradients’ directions and magnitudes. Peaks in this histogram indicate predominant directions. The formula incorporates the number of peaks and the distribution of these peaks to determine the Taruma Directionality value. A higher value suggests a stronger predominant directionality, while a lower value indicates a more balanced distribution of directions. The Taruma Directionality metric is valuable for characterizing the orientation and alignment of features in an image. It provides insights into the image's structural patterns and can be used in applications such as texture analysis, pattern recognition, and image classification.

The Taruma Coarseness metric is calculated based on the wavelet transform coefficients of an image. These coefficients capture variations in texture and patterns across different scales. Let $C_{wavelet}\;$denote the set of wavelet transform coefficients of the image, the Taruma Coarseness is calculated as the normalized sum of the absolute values of these coefficients $C{}_{taruma}^{\prime }\;=\;\sum c_{wavelet}/N$, where N is the image size or the total number of pixels in the image. The Taruma Coarseness metric provides a quantified measure of the roughness or coarseness of an image's texture. A higher value indicates a greater presence of coarse or rough textures, while a lower value suggests a smoother and finer texture. This metric is particularly useful for characterizing textures in images and can be employed in various applications, such as image analysis and classification tasks.

The Taruma Linelikeness metric quantifies the presence and prevalence of linear patterns or lines in an image. It involves analyzing the image's gradient components and direction to determine the extent to which the image contains linear features. The Taruma Linelikeness is computed using $L_{taruma}$ indicates a stronger prevalence of linear features, while a lower value suggests a more balanced distribution of orientations. The Taruma Linelikeness metric is useful for identifying and characterizing linear patterns in images. It can be applied in various contexts, such as line detection, texture analysis, and pattern recognition tasks. The Taruma Regularity metric quantifies the uniformity and repeatability of patterns present in an image. It involves analyzing the properties of the image's co-occurrence matrix, which captures the relationships between pixel intensities at various distances and angles. The Taruma Regularity is computed using the following formula: $R_{taruma}\;=\;\left[1\;+\;Contrast\;-\;Correlation\right]$, where Contrast is a measure of the intensity contrast between neighboring pixel pairs in the co-occurrence matrix and Correlation quantifies the linear dependence between pixel pairs in the matrix. The formula combines these two properties to calculate the regularity of patterns in the image. A higher value of $R_{taruma}$ indicates a higher degree of regularity and uniformity in patterns, while a lower value suggests more irregular or varied patterns.

**Fig. 3:**
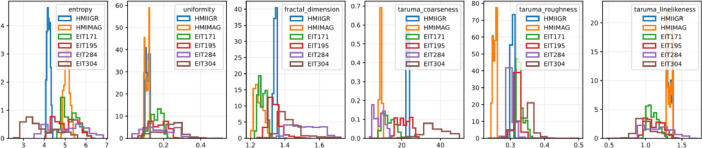
Normalized histogram of some of the parameters calculated in the data set constructed for entropy, uniformity, fractal dimension, taruma coarseness, taruma roughness, taruma linelikeness, considering all the years from 2011 to 2023.

The Taruma Roughness metric quantifies the degree of roughness or texture irregularity present in an image. It involves analyzing the image's texture properties using the co-occurrence matrix, which captures the relationships between pixel intensities at various distances and angles. The Taruma Roughness is computed using the following formula: $R{}_{taruma}^{\prime }\;=\;\left(1\;-\;Energy\right)\;\times \;Homogenety$, where Energy is a measure of the distribution of pixel pair intensities in the co-occurrence matrix and Homogeneity quantifies the similarity of pixel pair intensities in the matrix. The formula combines these two properties to calculate the roughness of textures in the image. A higher value of $R{}_{taruma}^{\prime }$ indicates a higher degree of roughness or texture irregularity, while a lower value suggests smoother and more uniform textures. The Taruma Roughness metric is useful for characterizing the roughness or fine-scale texture variations in images. It can be applied in tasks such as texture analysis, quality assessment, and image enhancement, where understanding the texture properties is important. Fig. 3 displays the normalized histogram of some parameters obtained during the dataset construction, considering all the available years. A distinct behavior is evident between the data from HMIIGR and HMIMAG images and those obtained from the extreme ultraviolet bands.

Finally, investigation in spectral and topological feaures in the context of spatial climate research is ultimately important for understanding various phenomena and establishing patterns for predictability and forecasting of solar wind and cosmic ray time series [[7], [8]].

## 4 Limitations

Not applicable.

## Ethics Statement

This work meets the requirements of ethics as stated in (https://www.elsevier.com/journals/data-in-brief/2352-3409/guide-for-authors) and (https://www.elsevier.com/about/policies/publishing-ethics#Authors). This work also does not involve studies with animals and humans.

## CRediT authorship contribution statement

**M. Tarazona-Alvarado:** Conceptualization, Methodology, Software, Writing – review & editing. **D. Sierra-Porta:** Conceptualization, Methodology, Software, Writing – review & editing.

## Declaration of Competing Interest

The authors declare that they have no known competing financial interests or personal relationships that could have appeared to influence the work reported in this paper.

## Data Availability

Dataset: Sun dynamics from topological features extraction (Original data) (Mendeley Data)
